# A pharmacovigilance study on clinical factors of active vitamin D_3_ analog-related acute kidney injury using the Japanese Adverse Drug Event Report Database

**DOI:** 10.1038/s41598-024-72505-w

**Published:** 2024-09-12

**Authors:** Yuki Kawai, Kazushi Uneda, Satoshi Miyata, Ayana Kunii, Shohei Nagayama, Kenji Baba, Tamio Iwamoto

**Affiliations:** 1 Teikyo University Graduate School of Public Health, 2-11-1 Kaga, Itabashi-Ku, Tokyo, 173-8605 Japan; 2 Department of Kampo Medicine, Aizu Medical Center, Fukushima Medical University, Aizuwakamatsu, Japan; 3 Department of Nephrology and Hypertension, Saiseikai Yokohamashi Nanbu Hospital, Yokohama, Japan

**Keywords:** Acute kidney injury, Active vitamin D_3_ analog, Eldecalcitol, Alfacalcidol, Calcitriol, Medical research, Nephrology, Risk factors

## Abstract

Acute kidney injury (AKI) due to vitamin D therapy for osteoporosis is encountered in clinical practice, but epidemiological studies are scarce. We aimed to determine the association between AKI and vitamin D therapy and to identify risk factors for AKI using the Japanese Adverse Drug Event Report database. We used reporting odds ratios (RORs) to detect signals and evaluate risk factors using multiple logistic regression analysis. Among 298,891 reports from April 2004 to September 2023, 1071 implicated active vitamin D_3_ analogs as suspect drugs for adverse events. There was a significant association between AKI and active vitamin D_3_ analogs (ROR [95% confidence interval {CI}], eldecalcitol: 16.75 [14.23–19.72], *P* < 0.001; alfacalcidol: 5.29 [4.07–6.87], *P* < 0.001; calcitriol: 4.46 [1.88–10.59], *P* < 0.001). The median duration of administration before AKI onset was 15.4 weeks. Multiple logistic regression analysis showed a significant association between AKI and age ≥ 70 years (odds ratio [95% CI], 1.47 [1.04–2.07]; *P* = 0.028), weight < 50 kg (1.55 [1.12–2.13]; *P* = 0.007), hypertension (1.90 [1.42–2.54]; *P* < 0.001), and concomitant use of nonsteroidal anti-inflammatory drugs (1.58 [1.10–2.25], *P* = 0.012) and magnesium oxide (1.96 [1.38–2.78]; *P* < 0.001). Our results suggest that active vitamin D_3_ analogs are associated with AKI development. Physicians prescribing these medications to patients with risk factors should consider the possibility of AKI, especially during the first 6 months.

## Introduction

Although the estimated incidence rate of acute kidney injury (AKI) in the general population has not been established, 9.6–12.2% of hospitalized patients are reported to develop AKI^1,2^. The incidence of AKI is increasing worldwide^3^. Additionally, 19–25% of AKI is induced in association with medications^4,5^. AKI may be a risk factor for long-term complications, such as end-stage renal failure and mortality^6,7^. Therefore, identifying clinical factors associated with the occurrence of AKI for individual medications is important for implementing preventive strategies in the clinical setting.

Although AKI due to vitamin D therapy, a treatment for osteoporosis, is often encountered in clinical practice, research on this topic is limited to case reports^8–10^ and case series^11–15^. There have been few epidemiological studies on AKI due to vitamin D therapy. Vitamin D drugs may induce hypercalcemia by promoting intestinal calcium absorption^16–19^. Although the pathogenesis of AKI owing to hypercalcemia is not fully understood, the following mechanisms have been reported: (1) circulating volume depletion occurs as a result of hypercalcemia, causing impaired renal concentrating capacity due to inhibition of sodium and chloride reabsorption in the ascending limb of Henle’s loop and a reduced response of antidiuretic hormone in collecting ducts^20,21^; (2) direct renal vasoconstrictive effects due to hypercalcemia^22^; and (3) tubulointerstitial injury due to calcium deposition^23,24^.

Vitamin D drugs are not generally the first choice for treating osteoporosis. However, sufficiency of vitamin D is a prerequisite for treating osteoporosis according to the National Osteoporosis Federation guidelines^25^. Furthermore, osteoporosis is still treated with vitamin D drugs not only because of vitamin D deficiency but also to avoid side effects such as osteonecrosis of the jaw with bisphosphonates.

Information on the occurrence of AKI associated with the three vitamin D drugs covered by insurance in Japan is primarily focused on eldecalcitol, which was launched in 2011; reports on the association with the other two drugs are scarce. Furthermore, a post-marketing observational study conducted 1 year after the launch of eldecalcitol reported a relatively low incidence of renal impairment adverse events (AEs) at 0.27%^26^. Additionally, clinical factors related to the occurrence of AKI as an AE with use of vitamin D drugs remain unknown.

Spontaneous reporting systems of adverse drug reactions, including the Japanese Adverse Drug Event Report (JADER) database and US Food and Drug Administration (FDA) adverse event reporting system (FAERS) have been used in pharmacovigilance analyses^27–31^. These databases are useful in detecting rare AEs or revealing new AEs by identifying drug-reaction pairs that occur with a significant disproportion compared with all other pairs^32,33^.

In this study, we aimed to determine the association between AKI and vitamin D drugs and to identify the risk factors for developing AKI using a large nationwide database on AEs.

## Results

### Data extraction and patients’ characteristics

Among 298,891 cases in the final analysis, there were 1071 cases of AEs with active vitamin D_3_ analog use, including 621 cases for eldecalcitol, 408 cases for alfacalcidol, and 42 cases for calcitriol. Of these 1071 cases, 309 cases involved AKI, among which 236 cases involved the use of eldecalcitol, 67 the use of alfacalcidol, and 6 the use of calcitriol. Table 1 shows a comparison of patient characteristics between those with AKI and those with other AEs associated with active vitamin D_3_ analog use. Cases with AKI tended to involve a higher proportion of female individuals than cases with other AEs. Additionally, cases of AKI included a significantly higher proportion of older patients (≥ 70 years) and those with low body weight (< 50 kg) (*P* < 0.001) than cases of other AEs. Cases of AKI included a significantly higher proportion of patients with a medical history of hypertension than cases of other AEs (*P* < 0.001); there was no difference in CKD, diabetes, or heart failure between the two groups. Additionally, higher proportions of concomitant NSAID and RASI use were observed in cases of AKI than in cases of other AEs (both *P* = 0.001). Similarly, cases of AKI included a significantly higher proportion of concomitant use of magnesium oxide than those involving other AEs (*P* < 0.001).

**Table 1:** Comparison of patients’ characteristics between those with AKI and those with other adverse events associated with active vitamin D_3_ analog use.

Variable	AKI (n = 309)	Other AEs (n = 762)	*P* value
Female, n (%)	269 (87.1)	628 (82.4)	0.062
Age (years), n (%)			< 0.001
< 70	61 (19.7)	251 (32.9)	
≥ 70	248 (80.3)	511 (67.1)	
Body weight, (kg), n (%)			< 0.001
< 50	219 (70.9)	439 (57.6)	
≥ 50	90 (29.1)	323 (42.4)	
Medical history, n (%)			
Chronic kidney disease	59 (19.1)	121 (15.9)	0.20
Diabetes	43 (13.9)	103 (13.5)	0.86
Hypertension	158 (51.1)	258 (33.9)	< 0.001
Heart failure	20 (6.5)	54 (7.1)	0.72
Concomitant use of drugs, n (%)			
NSAIDs	69 (22.3)	105 (13.8)	0.001
RASIs	58 (18.8)	84 (11.0)	0.001
Loop diuretics	32 (10.4)	79 (10.4)	1.00
Thiazide and thiazide-related diuretics	12 (3.9)	19 (2.5)	0.22
Magnesium oxide	75 (24.3)	99 (13.0)	< 0.001

*AKI* acute kidney injury, *AE* adverse event, *NSAIDs* nonsteroidal anti-inflammatory drugs, *RASIs* renin–angiotensin system inhibitors.

### AKI signal detection

Active vitamin D_3_ analogs showed a significant signal associated with AKI (ROR = 11.16; 95% CI = 9.76–12.75; *P* < 0.001). Furthermore, there was a significant association between AKI and each active vitamin D_3_ analog (ROR [95% CI], eldecalcitol: 16.75 [14.23–19.72], *P* < 0.001; alfacalcidol: 5.29 [4.07–6.87], *P* < 0.001; calcitriol: 4.46 [1.88–10.59], *P* < 0.001).

### Time to AKI onset

**Fig. 1:**
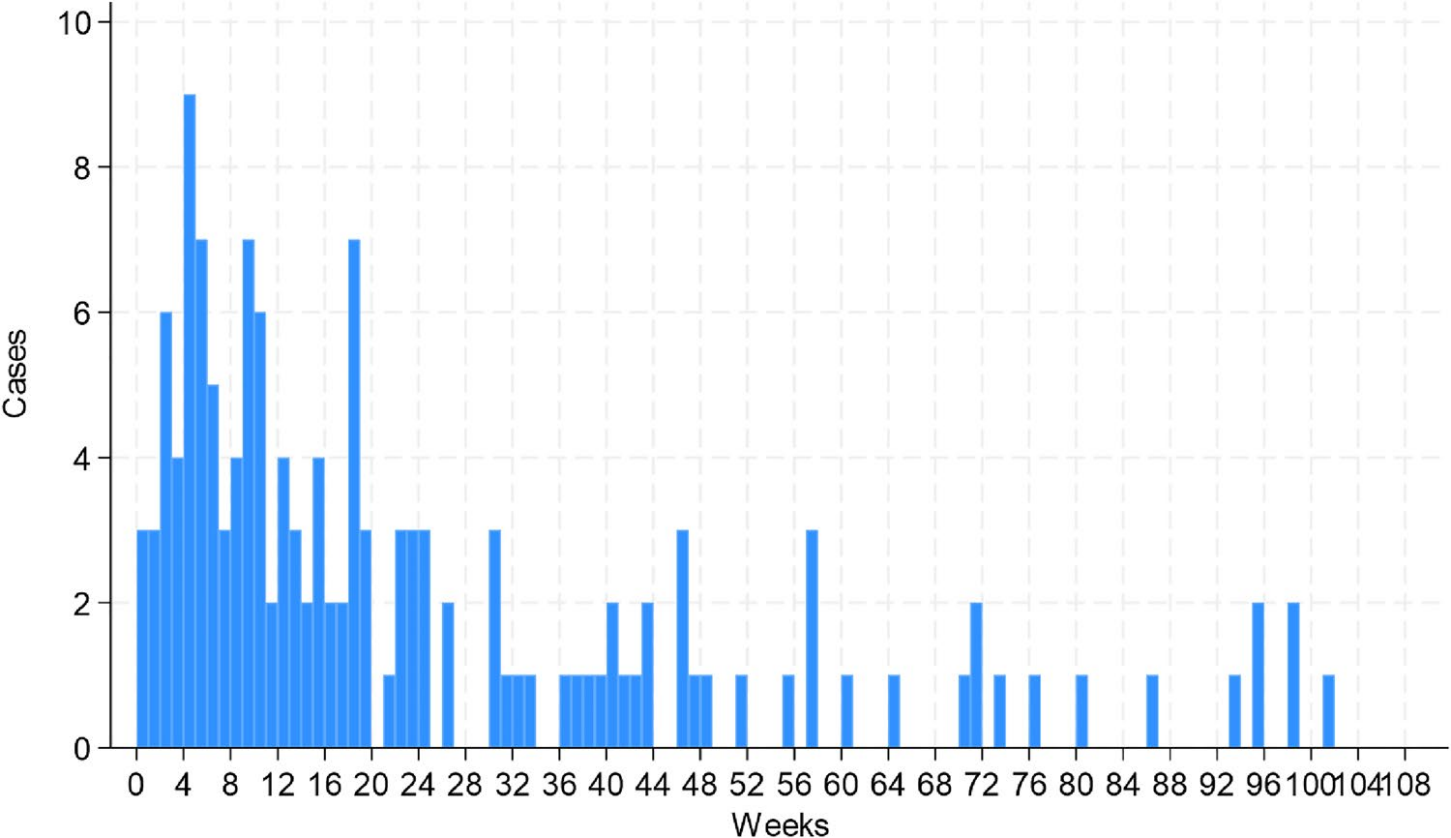
Histogram of the number of weeks from the date of initiating active vitamin D_3_ analogs to the date of onset of adverse events of acute kidney injury.

Figure 1 shows a histogram of the number of days from the date of initiating an active vitamin D_3_ analog to the date of AKI onset. The median duration of vitamin D_3_ analog administration before AKI onset was 15.4 (interquartile range [IQR] 6.5–35.1) weeks. The median durations for eldecalcitol, alfacalcidol, and calcitriol were 15.4 (IQR 6.7–35.9), 15.6 (IQR 9.4–24.3), and 14.9 (IQR 4.8–28.6) weeks, respectively.

### Logistic regression analysis of risk factors for active vitamin D_3_ analog-related AKI

Table 2 shows the results of multiple logistic regression analysis for factors associated with AKI in users of active vitamin D_3_ analogs. There was a significant association between AKI and age ≥ 70 years (OR [95% CI], 1.47 [1.04–2.07]; *P* = 0.028), body weight < 50 kg (1.55 [1.12–2.13]; *P* = 0.007), hypertension (1.90 [1.42–2.54]; *P* < 0.001), and concomitant use of NSAIDs (1.58 [1.10–2.25], *P* = 0.012) and magnesium oxide (1.96 [1.38–2.78]; *P* < 0.001). In the logistic model, the area under the receiver operating characteristic curve was 0.67 (95% CI 0.63–0.70), and the Hosmer–Lemeshow goodness-of-fit test showed *P* = 0.73.

**Table 2:** Multiple logistic regression analysis of factors associated with AKI in users of active vitamin D_3_ analogs.

	Univariate analysis		Multivariate analysis	
Variable	OR (95% CI)	*P* value	OR (95% CI)	*P* value
Sex (female)	1.43 (0.98–2.10)	0.063	1.14 (0.74–1.73)	0.56
Age (≥ 70)	2.00 (1.45–2.75)	< 0.001	1.47 (1.04–2.07)	0.028
Body weight (< 50),	1.79 (1.35–2.38)	< 0.001	1.55 (1.12–2.13)	0.007
Chronic kidney disease	1.25 (0.89–1.76)	0.20	1.29 (0.88–1.89)	0.18
Diabetes	1.03 (0.70–1.52)	0.86	0.89 (0.59–1.35)	0.59
Hypertension	2.04 (1.56–2.67)	< 0.001	1.90 (1.42–2.54)	< 0.001
Heart failure	0.91 (0.53–1.54)	0.72	0.72 (0.40–1.30)	0.28
NSAIDs	1.80 (1.28–2.52)	0.001	1.58 (1.10–2.25)	0.012
RASIs	1.87 (1.30–2.69)	0.001	1.35 (0.91–2.01)	0.14
Loop diuretics	1.00 (0.65–1.54)	1.00	0.89 (0.55–1.44)	0.63
Thiazide and thiazide-related diuretics	1.58 (0.76–3.30)	0.22	1.08 (0.49–2.37)	0.85
Magnesium oxide	2.15 (1.54–3.00)	< 0.001	1.96 (1.38–2.78)	< 0.001

*AKI* acute kidney injury, *CI* confidence interval, *NSAIDs* nonsteroidal anti-inflammatory drugs, *OR* odds ratio, *RASIs* renin–angiotensin system inhibitors.

## Discussion

To the best of our knowledge, this is the first study to assess the risk of developing AKI in vitamin D drug users and to investigate associated clinical factors using a nationwide large-scale database. Eldecalcitol, alfacalcidol, and calcitriol showed significant signals associated with the development of AKI. Furthermore, older age (≥ 70 years), low body weight (< 50 kg), a medical history of hypertension, and concomitant use of NSAIDs and magnesium oxide were risk factors associated with the development of AKI in active vitamin D_3_ analog users with AE reported to PMDA.

The association of older age and low body weight with AKI in users of active vitamin D_3_ analogs is consistent with findings in previous case series^11,12,14^. Aging degenerates the kidney structure and reduces renal autoregulatory capacity, which increases susceptibility to acute injury^34^. Specifically, renal vasoconstriction under hypercalcemia may be more likely in older patients because of a high sensitivity to vasoconstriction factors, leading to a subsequent severe decrease in the glomerular filtration rate^35^. Another change in the aging kidney is decreased renal tubular sodium reabsorption^36^. Additionally, because of a decrease in total body water^37^, older patients may be less tolerant to circulating volume depletion under hypercalcemia. Although previous studies have suggested that obesity is a potential risk factor for AKI in several clinical situations^38^, we showed that low body weight was a risk factor for active vitamin D_3_ analog-related AKI. Sensitivity to volume depletion under hypercalcemia could enhance the onset of AKI because patients with a low body weight have less total volume.

In this study, hypertension, as well as the concomitant use of NSAIDs and magnesium oxide, were significantly associated with developing AKI. The association between hypertension and the development of AKI has been reported in populations including older people^39^ and patients undergoing cardiothoracic surgery^40^ or transcatheter aortic valve implantation^41^. Hypertension may predispose to AKI under hypercalcemia because it causes decreased autoregulation of renal blood flow^42,43^ as well as renal arteriosclerosis and tubulointerstitial injury^44,45^. NSAIDs attenuate renal vasodilation and cause AKI by indirectly suppressing the production of prostaglandins via the inhibition of cyclooxygenase^46,47^. If the circulating blood volume is reduced owing to hypercalcemia, NSAIDs may induce a marked reduction in renal blood flow, increasing the likelihood of AKI occurrence. Magnesium oxide is reported to be associated with higher blood calcium levels in patients hospitalized with vitamin D-induced AKI^11^ Magnesium oxide intake can lead to metabolic alkalosis and increased renal tubular calcium reabsorption, potentially resulting in AKI^48,49^.

RASIs, loop diuretics, thiazide and thiazide-related diuretics, CKD, diabetes, and heart failure were not associated with developing AKI in the present study. Although we predicted that RASIs would induce AKI under hypercalcemia, similar to its induction under hypovolemia^50^, we found no association between active vitamin D_3_ analogs and AKI. Contrary to our predictions, RASIs might have offset the increased risk of AKI under hypercalcemia in this study, considering its reported potential to protect against AKI in patients aged 75 years and older^39^ and in preoperative patients^51,52^. Additionally, because loop diuretics increase urinary calcium excretion and are also used with saline to treat hypercalcemia^53^, we expected them to show a negative association with AKI. However, although loop diuretics may improve hypercalcemia, as mentioned above, they also may contribute to AKI by reducing circulating volume. Therefore, the lack of an association between loop diuretics and AKI in the present study might be attributed to these dual effects. Furthermore, thiazide and thiazide-related diuretics, which decrease urinary calcium excretion^53^, as well as CKD, heart failure, and diabetes, showed no association with AKI. Further investigation is required to determine their mechanism of action.

Although female sex was significantly associated with AKI in univariate logistic regression analysis, this significance was lost in multivariate analysis. There is limited research on sex differences in the risk of AKI, and the association between sex and AKI varies according to previous reports. Several reports have suggested that drug-induced AKI poses a higher risk in female individuals^37,54^ whereas other reports have shown that male hospitalized patients have a higher risk of AKI^55,56^. The possibility of female users of active vitamin D_3_ analog having a higher risk of AKI onset has been previously discussed^11^. This higher risk in female individuals may be owing to the higher prevalence of osteoporosis in women^57^ and the corresponding larger proportion of female users of vitamin D_3_ analogs.

In this study, the median duration of vitamin D_3_ analog administration prior to AKI onset was 15.4 weeks. In a previous case series^14^, the average duration of vitamin D_3_ administration was 9 weeks. Because that case series included cases involving intramuscular injection in addition to oral administration, the difference in the route of vitamin D administration between that case series and the present study may have affected the difference in duration before AKI onset between studies.

A strength of this study is the identification of clinical factors associated with AKI in active vitamin D_3_ analog users using a large national database. Although AKI caused by vitamin D drugs is frequently experienced in clinical practice, few epidemiological studies have examined the factors involved in this association. Therefore, the clinical factors suggested in previous case reports and case series were assessed in this study.

This study has several limitations. First, because the JADER database only includes cases in which AEs occurred, we were unable to include information on patients in whom no AEs occurred. Therefore, we could not calculate the incidence of AKI. Second, because the JADER database comprises spontaneous reports of AEs, several biases, such as reporting bias, underreporting, and missing data, may have been present and affected the RORs. Therefore, although the ROR is a well-established indicator in pharmacovigilance studies^58^, comparing the risk of AEs between different drugs based solely on ROR values may be unsuitable. Furthermore, because alfacalcidol and calcitriol are also prescribed for other diseases, including hypoparathyroidism, the drug indication might affect our results. Third, the JADER database only includes AEs occurring in Japan; therefore, it may be difficult to extrapolate the findings of this study to populations in other countries. Fourth, the risk of AKI associated with vitamin D_3_ analogs covered by insurance in Japan for osteoporosis treatment in this study could potentially be overestimated owing to the following factors: (1) the types of vitamin D drugs for osteoporosis frequently used in Japan and other countries differ; (2) the diagnostic criteria for osteoporosis in Japan^59^ not only include the T-score based on the World Health Organization definition^60^ but also young adult mean values and the presence of fractures, which could potentially lead to the initiation of treatment. Additionally, warnings were issued by the PMDA for one vitamin D_3_ analog regarding hypercalcemia. Therefore, the findings of this study may need to be validated using overseas databases, such as the FAERS. Fifth, the clinical background information on patients that was available in the JADER database was limited. This might have affected the results with poor calibration and discrimination based on logistic regression, indicating the presence of other risk factors associated with the occurrence of AKI in vitamin D users. Sixth, female patients predominated in both groups in our study. This predominance might have affected our regression results. Seventh, in view of the finding that magnesium oxide is a potential factor for AKI, the AKI might be due to hypercalcemia. However, since the JADER database is secondary data, it was hard to include blood calcium levels and the accurate doses of active vitamin D_3_ analogs in our analysis. Verifying the results of this study using electronic health care databases, including claims databases and electronic medical records databases that include high-volume data and high-quality information, may address these limitations.

In conclusion, the results of this study suggest that active vitamin D_3_ analogs may increase the risk of AKI. When administering active vitamin D_3_ analogs to patients with specific characteristics, such as older age, low body weight, hypertension, and concomitant use of NSAIDs and magnesium oxide, careful attention should be paid to acute deterioration of renal function, especially during the first 6 months.

## Methods

### Data source

We used the JADER database in this study. This database is an anonymized, open-access repository provided on the Pharmaceuticals and Medical Devices Agency (PMDA) website^61^. The JADER database contains spontaneous reports of drug-related AEs in Japan since April 2004. This database comprises four data tables: DEMO (patient demographic information), DRUG (drug information), REAC (AE information), and HIST (medical history). In the DRUG table, the involvement of drugs in AEs is categorized into three categories of suspected drugs, concomitant drugs, and interacting drugs.

### Data collection

**Fig. 2:**
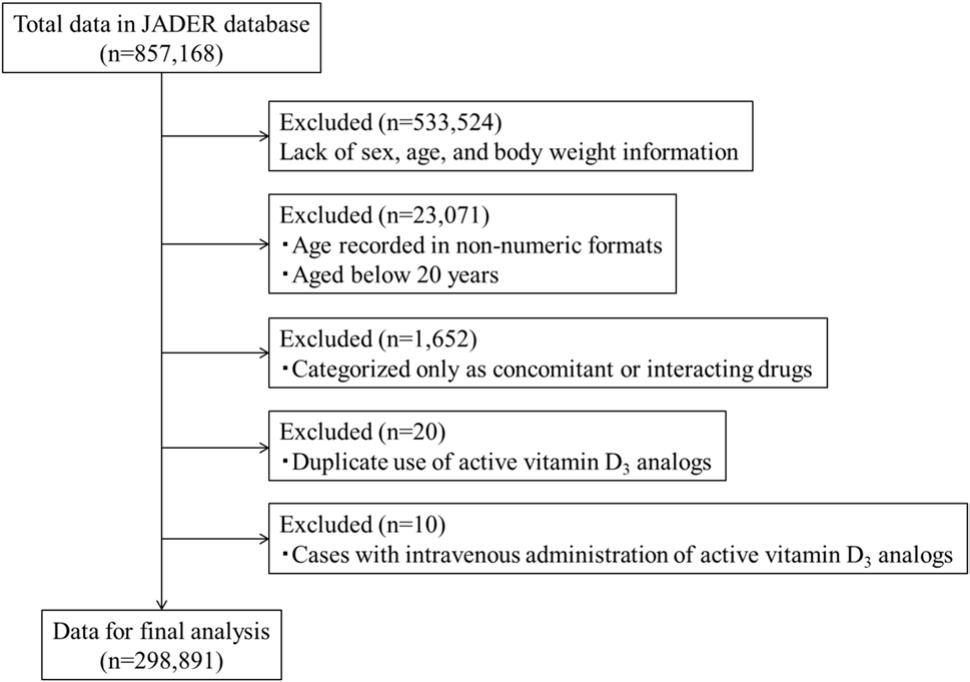
Flowchart of the construction of the dataset used in our study. *JADER* Japanese Adverse Drug Event Report.

Data from April 2004 to September 2023 were downloaded from the PMDA website. On the basis of previous reports^62–64^, cases aged < 20 years, those with missing or unknown information on sex, age, and body weight, and those with age recorded in non-numeric formats (e.g., pediatric, adult, and older), were excluded. We also excluded cases categorized only as having concomitant or interacting drugs, those with intravenous administration of active vitamin D_3_ analogs, and those with duplicate use of active vitamin D_3_ analogs. Among 857,168 cases, the final analysis included data of 298,891 cases (Fig. 2).

### Definition of target and concomitant drugs

Active vitamin D_3_ analogs, nonsteroidal anti-inflammatory drugs (NSAIDs), renin–angiotensin system inhibitors (RASIs; e.g., angiotensin receptor blockers and angiotensin-converting enzyme inhibitors), loop diuretics, thiazide and thiazide-related diuretics, and magnesium oxide were defined according to the drug names in the Kyoto Encyclopedia of Genes and Genomes drug database^65^, with reference to a previous report^64^. For the purpose of this study, vitamin D_3_ drugs were limited to active vitamin D_3_ analogs that are covered by insurance for the treatment of osteoporosis. Aspirin is classified as an NSAID in the Kyoto Encyclopedia of Genes and Genomes drug database. However, because aspirin is administered as an antiplatelet agent, it was not included in the category of NSAIDs in this study. The generic names of all drugs used in the analysis are shown in Supplementary Table 1.

### Definition of AEs and comorbidities

AEs and comorbidities in the JADER database are coded using preferred terms (PTs) in the Medical Dictionary for Regulatory Activities (MedDRA)/Japanese version 26.1. When searching for specific diseases in the JADER database, we used standardized MedDRA Queries (SMQs), which groups MedDRA terms, generally consisting of PT-level terms related to a defined medical condition or area of interest^66^. AKI was defined using the PTs classified as “acute renal failure” in the SMQs, as reported previously^27,64^. Similarly, CKD was identified using the PTs classified as “chronic kidney disease,” “kidney failure”, or “dialysis”, in accordance with previous studies^67,68^. Hypertension, diabetes, and heart failure were also defined using the PTs classified as “hypertension”, “hyperglycemia/new onset diabetes mellitus”, and “cardiac failure”, respectively. PTs used for the identification of each disease are shown in Supplementary Table 2.

### Definition of time to onset of AKI

The time to onset of AKI was calculated for reports of AKI AEs due to use of active vitamin D_3_ analogs in which the date of onset of the AE and date of initiating administration were listed in years, months, days, or years and months, as previously reported^69,70^. The time to onset of AEs used in the analysis was limited to 730 days (2 years). The number of days to onset of AEs was calculated as follows: (date of AE onset) − (date of starting administration) + 1^70^.

### Statistical analysis

We conducted disproportionality analysis to detect target drug-related AKI signals. We calculated the reporting odds ratio (ROR) using a two-by-two contingency table, as described previously^71,72^. In this study, we chose the full dataset comparator for signal detection^73^ because this comparator reportedly has the advantage of reducing the impact of reporting biases compared with restricted comparators, including an active comparator. The calculation method of RORs and 95% confidence intervals (CIs) is shown in Supplementary Table 3. A significant signal was detected when the lower limit of the 95% CI for the ROR was > 1. Multiple logistic regression using the forced-entry method was conducted to identify the factors associated with AKI among users of active vitamin D_3_ analogs. The covariates included sex^11^, age (< 70 or ≥ 70 years)^11,14^, body weight (< 50 or ≥ 50 kg)^12^, chronic kidney disease (CKD)^14^, and concomitant use of NSAIDs^11,12^, RASIs^11,12^, loop diuretics^11^, thiazide and thiazide-related diuretics, and magnesium oxide^11^, which are reported or considered to be associated with the onset of AKI with vitamin D therapy. Moreover, hypertension, diabetes, and heart failure were included as well-known risk factors for AKI^39,63,74,75^. We assessed the discrimination and calibration of the logistic model using the area under the receiver operating characteristic curve and the Hosmer–Lemeshow goodness-of-fit test, respectively. Two-sided *P* values < 0.05 were considered statistically significant. Statistical analyses were performed using R version 4.2.2 (R Foundation for Statistical Computing, Vienna, Austria) and Stata BE version 18.0 (StataCorp, College Station, TX, USA).

## Supplementary Information

Supplementary Tables.

## Data Availability

The datasets generated and analyzed during the current study are available from the corresponding author on reasonable request.

## References

[1] Al-Jaghbeer, M., Dealmeida, D., Bilderback, A., Ambrosino, R. & Kellum, J. A. Clinical decision support for in-hospital AKI. *J. Am. Soc. Nephrol.* **29**, 654–660. 10.1681/asn.2017070765 (2018). 29097621 10.1681/asn.2017070765 PMC5791078

[2] Lafrance, J. P. & Miller, D. R. Acute kidney injury associates with increased long-term mortality. *J. Am. Soc. Nephrol.* **21**, 345–352. 10.1681/asn.2009060636 (2010). 20019168 10.1681/asn.2009060636 PMC2834549

[3] Hoste, E. A. J. *et al.* Global epidemiology and outcomes of acute kidney injury. *Nat. Rev. Nephrol.* **14**, 607–625. 10.1038/s41581-018-0052-0 (2018). 30135570 10.1038/s41581-018-0052-0

[4] Uchino, S. *et al.* Acute renal failure in critically ill patients: A multinational, multicenter study. *JAMA* **294**, 813–818. 10.1001/jama.294.7.813 (2005). 16106006 10.1001/jama.294.7.813

[5] Mehta, R. L. *et al.* Spectrum of acute renal failure in the intensive care unit: The PICARD experience. *Kidney Int.* **66**, 1613–1621. 10.1111/j.1523-1755.2004.00927.x (2004). 15458458 10.1111/j.1523-1755.2004.00927.x

[6] Coca, S. G., Yusuf, B., Shlipak, M. G., Garg, A. X. & Parikh, C. R. Long-term risk of mortality and other adverse outcomes after acute kidney injury: A systematic review and meta-analysis. *Am. J. Kidney Dis.* **53**, 961–973. 10.1053/j.ajkd.2008.11.034 (2009). 19346042 10.1053/j.ajkd.2008.11.034 PMC2726041

[7] See, E. J. *et al.* Long-term risk of adverse outcomes after acute kidney injury: A systematic review and meta-analysis of cohort studies using consensus definitions of exposure. *Kidney Int.* **95**, 160–172. 10.1016/j.kint.2018.08.036 (2019). 30473140 10.1016/j.kint.2018.08.036

[8] De Vincentis, S. *et al.* How much vitamin D is too much? A case report and review of the literature. *Endocr. Metab. Immune Disord. Drug Targets* **21**, 1653–1659. 10.2174/1871530320666201007152230 (2021). 33030138 10.2174/1871530320666201007152230 PMC8811610

[9] Guerra, V., Vieira Neto, O. M., Laurindo, A. F., Paula, F. J. & Moysés Neto, M. Hypercalcemia and renal function impairment associated with vitamin D toxicity: Case report. *J. Bras. Nefrol.* **38**, 466–469. 10.5935/0101-2800.20160074 (2016). 28001186 10.5935/0101-2800.20160074

[10] Hemachandar, R., Shanmugam, L., Malepati, B. & Venugopal, S. Hyper vitaminosis D: Are we overprescribing vitamin D?. *J. Fam. Med. Prim. Care* **3**, 464–466. 10.4103/2249-4863.148153 (2014). 10.4103/2249-4863.148153 PMC4311368 25657969

[11] Aihara, S. *et al.* Hypercalcemia and acute kidney injury induced by eldecalcitol in patients with osteoporosis: A case series of 32 patients at a single facility. *Ren. Fail.* **41**, 88–97. 10.1080/0886022x.2019.1578667 (2019). 30909788 10.1080/0886022x.2019.1578667 PMC6442105

[12] Narisue, M. *et al.* Survey of prescriptions for triple whammy drug combinations with vitamin D as a possible fourth whammy. *Int. J. Clin. Pharmacol. Ther.* **61**, 8–15. 10.5414/cp204234 (2023). 36373327 10.5414/cp204234

[13] Wani, M., Wani, I., Banday, K. & Ashraf, M. The other side of vitamin D therapy: A case series of acute kidney injury due to malpractice-related vitamin D intoxication. *Clin. Nephrol.* **86**(2016), 236–241. 10.5414/cn108904 (2016). 27719737 10.5414/cn108904

[14] Chowdry, A. M., Azad, H., Najar, M. S. & Mir, I. Acute kidney injury due to overcorrection of hypovitaminosis D: A tertiary center experience in the Kashmir Valley of India. *Saudi J. Kidney Dis. Transplant.* **28**, 1321–1329. 10.4103/1319-2442.220873 (2017). 10.4103/1319-2442.220873 29265043

[15] Kaur, P., Mishra, S. K. & Mithal, A. Vitamin D toxicity resulting from overzealous correction of vitamin D deficiency. *Clin. Endocrinol. (Oxford)* **83**, 327–331. 10.1111/cen.12836 (2015). 10.1111/cen.12836 26053339

[16] Matsumoto, T., Takano, T., Yamakido, S., Takahashi, F. & Tsuji, N. Comparison of the effects of eldecalcitol and alfacalcidol on bone and calcium metabolism. *J. Steroid Biochem. Mol. Biol.* **121**, 261–264. 10.1016/j.jsbmb.2010.03.035 (2010). 20298784 10.1016/j.jsbmb.2010.03.035

[17] Uenishi, K., Tokiwa, M., Kato, S. & Shiraki, M. Stimulation of intestinal calcium absorption by orally administrated vitamin D3 compounds: A prospective open-label randomized trial in osteoporosis. *Osteoporos. Int.* **29**, 723–732. 10.1007/s00198-017-4351-2 (2018). 29273827 10.1007/s00198-017-4351-2 PMC5834567

[18] Tsukamoto, Y., Watanabe, T., Nakagami, T. & Morishita, K. Effect of treatment with oral calcitriol on calcium metabolism and fasting serum 25(OH)- or 1,25(OH)2-vitamin D level in Japanese postmenopausal women. *Endocr. J.* **50**, 681–687. 10.1507/endocrj.50.681 (2003). 14709838 10.1507/endocrj.50.681

[19] Tebben, P. J., Singh, R. J. & Kumar, R. Vitamin D-mediated hypercalcemia: Mechanisms, diagnosis, and treatment. *Endocr. Rev.* **37**, 521–547. 10.1210/er.2016-1070 (2016). 27588937 10.1210/er.2016-1070 PMC5045493

[20] Peterson, L. N., McKay, A. J. & Borzecki, J. S. Endogenous prostaglandin E2 mediates inhibition of rat thick ascending limb Cl reabsorption in chronic hypercalcemia. *J. Clin. Investig.* **91**, 2399–2407. 10.1172/jci116473 (1993). 8390479 10.1172/jci116473 PMC443298

[21] Renkema, K. Y. *et al.* The calcium-sensing receptor promotes urinary acidification to prevent nephrolithiasis. *J. Am. Soc. Nephrol.* **20**, 1705–1713. 10.1681/asn.2008111195 (2009). 19470676 10.1681/asn.2008111195 PMC2723980

[22] Levi, M., Ellis, M. A. & Berl, T. Control of renal hemodynamics and glomerular filtration rate in chronic hypercalcemia. Role of prostaglandins, renin-angiotensin system, and calcium. *J. Clin. Investig.* **71**, 1624–1632. 10.1172/jci110918 (1983). 6345587 10.1172/jci110918 PMC370368

[23] Graidis, S., Papavramidis, T. S. & Papaioannou, M. Vitamin D and acute kidney injury: A two-way causality relation and a predictive, prognostic, and therapeutic role of vitamin D. *Front. Nutr.* **7**, 630951. 10.3389/fnut.2020.630951 (2020). 33748167 10.3389/fnut.2020.630951 PMC7969500

[24] Rosen, S. *et al.* Hypercalcemic nephropathy: Chronic disease with predominant medullary inner stripe injury. *Kidney Int.* **37**, 1067–1075. 10.1038/ki.1990.87 (1990). 2342245 10.1038/ki.1990.87

[25] Cosman, F. *et al.* Clinician’s guide to prevention and treatment of osteoporosis. *Osteoporos. Int.* **25**, 2359–2381. 10.1007/s00198-014-2794-2 (2014). 25182228 10.1007/s00198-014-2794-2 PMC4176573

[26] Saito, H. *et al.* The safety and effectiveness profile of eldecalcitol in a prospective, post-marketing observational study in Japanese patients with osteoporosis: Interim report. *J. Bone Mineral Metab.* **35**, 456–463. 10.1007/s00774-016-0779-2 (2017). 10.1007/s00774-016-0779-2 27699492

[27] Nakao, S. *et al.* Pharmacovigilance study of anti-infective-related acute kidney injury using the Japanese adverse drug event report database. *BMC Pharmacol. Toxicol.* **22**, 47. 10.1186/s40360-021-00513-x (2021). 34462002 10.1186/s40360-021-00513-x PMC8404262

[28] Hosohata, K. *et al.* Surveillance of drugs that most frequently induce acute kidney injury: A pharmacovigilance approach. *J. Clin. Pharm. Ther.* **44**, 49–53. 10.1111/jcpt.12748 (2019). 30014591 10.1111/jcpt.12748

[29] Arai, M., Shirakawa, J., Konishi, H., Sagawa, N. & Terauchi, Y. Bullous pemphigoid and dipeptidyl peptidase 4 inhibitors: A disproportionality analysis based on the Japanese Adverse Drug Event Report Database. *Diabetes Care* **41**, e130–e132. 10.2337/dc18-0210 (2018). 30002201 10.2337/dc18-0210

[30] Wu, B. *et al.* Proton pump inhibitors associated acute kidney injury and chronic kidney disease: Data mining of US FDA adverse event reporting system. *Sci. Rep.* **11**, 3690. 10.1038/s41598-021-83099-y (2021). 33574396 10.1038/s41598-021-83099-y PMC7878877

[31] Welch, H. K., Kellum, J. A. & Kane-Gill, S. L. Drug-associated acute kidney injury identified in the United States food and drug administration adverse event reporting system database. *Pharmacotherapy* **38**, 785–793. 10.1002/phar.2152 (2018). 29883524 10.1002/phar.2152

[32] Poluzzi, E., Raschi, E., Piccinni, C. & Ponti, F. Data mining techniques in pharmacovigilance: Analysis of the publicly accessible FDA adverse event reporting system (AERS). *IntechOpen* **12**, 266–302. 10.5772/50095 (2012). 10.5772/50095

[33] Cutroneo, P. M. *et al.* Conducting and interpreting disproportionality analyses derived from spontaneous reporting systems. *Front. Drug Saf. Regul.* **3**, 1323057. 10.3389/fdsfr.2023.1323057 (2023). 10.3389/fdsfr.2023.1323057 PMC12443087 40980108

[34] Anderson, S. *et al.* Acute kidney injury in older adults. *J. Am. Soc. Nephrol.* **22**, 28–38. 10.1681/asn.2010090934 (2011). 21209252 10.1681/asn.2010090934

[35] Jerkić, M., Vojvodić, S. & López-Novoa, J. M. The mechanism of increased renal susceptibility to toxic substances in the elderly. Part I. The role of increased vasoconstriction. *Int. Urol. Nephrol.* **32**, 539–547. 10.1023/a:1014484101427 (2001). 11989542 10.1023/a:1014484101427

[36] Chang-Panesso, M. Acute kidney injury and aging. *Pediatr. Nephrol.* **36**, 2997–3006. 10.1007/s00467-020-04849-0 (2021). 33411069 10.1007/s00467-020-04849-0 PMC8260619

[37] Izzedine, H. & Perazella, M. A. Anticancer drug-induced acute kidney injury. *Kidney Int. Rep.* **2**, 504–514. 10.1016/j.ekir.2017.02.008 (2017). 29318217 10.1016/j.ekir.2017.02.008 PMC5720534

[38] Schiffl, H. & Lang, S. M. Obesity, acute kidney injury and outcome of critical illness. *Int. Urol. Nephrol.* **49**, 461–466. 10.1007/s11255-016-1451-4 (2017). 27822672 10.1007/s11255-016-1451-4

[39] Stille, K., Kribben, A. & Herget-Rosenthal, S. Incidence, severity, risk factors and outcomes of acute kidney injury in older adults: Systematic review and meta-analysis. *J. Nephrol.* **35**, 2237–2250. 10.1007/s40620-022-01381-2 (2022). 35932418 10.1007/s40620-022-01381-2

[40] Metz, L. I., LeBeau, M. E., Zlabek, J. A. & Mathiason, M. A. Acute renal failure in patients undergoing cardiothoracic surgery in a community hospital. *WMJ* **108**, 109–114 (2009). 19437938

[41] Bagur, R. *et al.* Acute kidney injury following transcatheter aortic valve implantation: Predictive factors, prognostic value, and comparison with surgical aortic valve replacement. *Eur. Heart J.* **31**, 865–874. 10.1093/eurheartj/ehp552 (2010). 20037180 10.1093/eurheartj/ehp552 PMC2848323

[42] Carlström, M., Wilcox, C. S. & Arendshorst, W. J. Renal autoregulation in health and disease. *Physiol. Rev.* **95**, 405–511. 10.1152/physrev.00042.2012 (2015). 25834230 10.1152/physrev.00042.2012 PMC4551215

[43] Almeida, J. B. *et al.* Severe hypertension induces disturbances of renal autoregulation. *Hypertension*10.1161/01.hyp.19.2_suppl.ii279 (1992). 1735593 10.1161/01.hyp.19.2_suppl.ii279

[44] Sommers, S. C., Relman, A. S. & Smithwick, R. H. Histologic studies of kidney biopsy specimens from patients with hypertension. *Am. J. Pathol.* **34**, 685–715 (1958). 13559400 PMC1934768

[45] Saltz, M., Sommers, S. C. & Smithwick, R. H. Clinicopathologic correlations of renal biopsies from essential hypertensive patients. *Circulation* **16**, 207–212. 10.1161/01.cir.16.2.207 (1957). 13447165 10.1161/01.cir.16.2.207

[46] Whelton, A. & Hamilton, C. W. Nonsteroidal anti-inflammatory drugs: Effects on kidney function. *J. Clin. Pharmacol.* **31**, 588–598. 10.1002/j.1552-4604.1991.tb03743.x (1991). 1894754 10.1002/j.1552-4604.1991.tb03743.x

[47] Schneider, V., Lévesque, L. E., Zhang, B., Hutchinson, T. & Brophy, J. M. Association of selective and conventional nonsteroidal antiinflammatory drugs with acute renal failure: A population-based, nested case-control analysis. *Am. J. Epidemiol.* **164**, 881–889. 10.1093/aje/kwj331 (2006). 17005625 10.1093/aje/kwj331

[48] Hanada, S., Iwamoto, M., Kobayashi, N., Ando, R. & Sasaki, S. Calcium-alkali syndrome due to vitamin D administration and magnesium oxide administration. *Am. J. Kidney Dis.* **53**, 711–714. 10.1053/j.ajkd.2008.11.015 (2009). 19185403 10.1053/j.ajkd.2008.11.015

[49] Vetter, T. & Lohse, M. J. Magnesium and the parathyroid. *Curr. Opinion Nephrol. Hypertens.* **11**, 403–410. 10.1097/00041552-200207000-00006 (2002). 10.1097/00041552-200207000-00006 12105390

[50] Ftouh, S. & Thomas, M. Acute kidney injury: Summary of NICE guidance. *BMJ* **347**, f4930. 10.1136/bmj.f4930 (2013). 23985310 10.1136/bmj.f4930

[51] Cheungpasitporn, W. *et al.* Preoperative renin-angiotensin system inhibitors use linked to reduced acute kidney injury: A systematic review and meta-analysis. *Nephrol. Dial. Transplant.* **30**, 978–988. 10.1093/ndt/gfv023 (2015). 25800881 10.1093/ndt/gfv023

[52] Chou, Y. H. *et al.* Associations between preoperative continuation of renin-angiotensin system inhibitor and cardiac surgery-associated acute kidney injury: A propensity score-matching analysis. *J. Nephrol.* **32**, 957–966. 10.1007/s40620-019-00657-4 (2019). 31595420 10.1007/s40620-019-00657-4

[53] Rose, B. D. Diuretics. *Kidney Int.* **39**, 336–352. 10.1038/ki.1991.43 (1991). 2002648 10.1038/ki.1991.43

[54] Perazella, M. A. Renal vulnerability to drug toxicity. *Clin. J. Am. Soc Nephrol* **4**, 1275–1283. 10.2215/cjn.02050309 (2009). 19520747 10.2215/cjn.02050309

[55] Neugarten, J., Golestaneh, L. & Kolhe, N. V. Sex differences in acute kidney injury requiring dialysis. *BMC Nephrol.* **19**, 131. 10.1186/s12882-018-0937-y (2018). 29884141 10.1186/s12882-018-0937-y PMC5994053

[56] Ebert, N. *et al.* Incidence of hospital-acquired acute kidney injury and trajectories of glomerular filtration rate in older adults. *BMC Nephrol.* **24**, 226. 10.1186/s12882-023-03272-5 (2023). 37528401 10.1186/s12882-023-03272-5 PMC10394866

[57] Yoshimura, N. *et al.* Prevalence of knee osteoarthritis, lumbar spondylosis, and osteoporosis in Japanese men and women: The research on osteoarthritis/osteoporosis against disability study. *J. Bone Mineral Metab.* **27**, 620–628. 10.1007/s00774-009-0080-8 (2009). 10.1007/s00774-009-0080-8 19568689

[58] Rothman, K. J., Lanes, S. & Sacks, S. T. The reporting odds ratio and its advantages over the proportional reporting ratio. *Pharmacoepidemiol. Drug Saf.* **13**, 519–523. 10.1002/pds.1001 (2004). 15317031 10.1002/pds.1001

[59] The committee for development of the guidelines on the prevention and treatment of osteoporosis. Guidelines on the prevention and treatment of osteoporosis 2015 (in Japanese). http://www.josteo.com/ja/guideline/doc/15_1.pdf. Accessed 11 Sep 2024.

[60] Kanis, J. A. Assessment of fracture risk and its application to screening for postmenopausal osteoporosis: Synopsis of a WHO report WHO study group. *Osteoporos. Int.* **4**, 368–381. 10.1007/bf01622200 (1994). 7696835 10.1007/bf01622200

[61] Pharmaceuticals and Medical Devices Agency. *Drug adverse event database: terms of use.* https://www.pmda.go.jp/safety/info-services/drugs/adr-info/suspected-adr/0003.html. Accessed 4 May 2024.

[62] Hatano, M. *et al.* Analysis of clozapine-induced seizures using the Japanese Adverse Drug Event Report database. *PLoS ONE* **18**, e0287122. 10.1371/journal.pone.0287122 (2023). 37307250 10.1371/journal.pone.0287122 PMC10259781

[63] Mitsuboshi, S., Kaseda, R. & Narita, I. Association between steroid use and nephropathy in patients who were administered a proton pump inhibitor: Analysis of the Japanese Adverse Event Report Database. *J. Clin. Pharmacol.* **62**, 272–275. 10.1002/jcph.1964 (2022). 34480763 10.1002/jcph.1964

[64] Kunitsu, Y. *et al.* Time until onset of acute kidney injury by combination therapy with “Triple Whammy” drugs obtained from Japanese Adverse Drug Event Report database. *PLoS ONE* **17**, e0263682. 10.1371/journal.pone.0263682 (2022). 35139129 10.1371/journal.pone.0263682 PMC8827454

[65] Kanehisa Laboratories*. KEGG: Kyoto Encyclopedia of Genes and Genomes.* https://www.genome.jp/kegg/. Accessed 4 May 2024.

[66] Medical Dictionary for Regulatory Activities*. Introductory Guide for Standardised MedDRA Queries (SMQs) Version 26.1.* https://admin.new.meddra.org/sites/default/files/guidance/file/SMQ_intguide_26_1_English.pdf. Accessed 4 May 2024.

[67] Yamada, T., Mitsuboshi, S., Suzuki, K., Nishihara, M. & Neo, M. Analysis of the frequency of ceftriaxone-induced encephalopathy using the Japanese Adverse Drug Event Report database. *Int. J. Clin. Pharm.* **44**, 1067–1071. 10.1007/s11096-022-01406-7 (2022). 35449346 10.1007/s11096-022-01406-7

[68] Yamada, T. *et al.* Risk of pregabalin-induced hypoglycemia: Analysis of the Japanese Adverse Drug Event Report Database. *J. Clin. Pharmacol.* **62**, 756–761. 10.1002/jcph.2009 (2022). 34817883 10.1002/jcph.2009

[69] Kanbayashi, Y. *et al.* Evaluation of lung adverse events with nivolumab using the spontaneous reporting system in Japan. *Sci. Rep.* **13**, 8819. 10.1038/s41598-023-35602-w (2023). 37258564 10.1038/s41598-023-35602-w PMC10232428

[70] Ando, G. *et al.* Evaluation of the expression time of Ganciclovir-induced adverse events using JADER and FAERS. *Biol. Pharm. Bulletin* **42**, 1799–1804. 10.1248/bpb.b19-00156 (2019). 10.1248/bpb.b19-00156 31685764

[71] Uneda, K. *et al.* Analysis of clinical factors associated with Kampo formula-induced pseudoaldosteronism based on self-reported information from the Japanese Adverse Drug Event Report database. *PLoS ONE* **19**, e0296450. 10.1371/journal.pone.0296450 (2024). 38165850 10.1371/journal.pone.0296450 PMC10760746

[72] van Puijenbroek, E. P. *et al.* A comparison of measures of disproportionality for signal detection in spontaneous reporting systems for adverse drug reactions. *Pharmacoepidemiol. Drug Saf.* **11**, 3–10. 10.1002/pds.668 (2002). 11998548 10.1002/pds.668

[73] Gravel, C. A. & Douros, A. Considerations on the use of different comparators in pharmacovigilance: A methodological review. *Br. J. Clin. Pharmacol.* **89**, 2671–2676. 10.1111/bcp.15802 (2023). 37226576 10.1111/bcp.15802

[74] Schefold, J. C., Filippatos, G., Hasenfuss, G., Anker, S. D. & von Haehling, S. Heart failure and kidney dysfunction: Epidemiology, mechanisms and management. *Nat. Rev. Nephrol.* **12**, 610–623. 10.1038/nrneph.2016.113 (2016). 27573728 10.1038/nrneph.2016.113

[75] Hapca, S. *et al.* The relationship between AKI and CKD in patients with type 2 diabetes: An observational cohort study. *J. Am. Soc. Nephrol.* **32**, 138–150. 10.1681/asn.2020030323 (2021). 32948670 10.1681/asn.2020030323 PMC7894655

