# Supplementary material for: A pharmacovigilance study on clinical factors of active vitamin D3 analog-related acute kidney injury using the Japanese Adverse Drug Event Report Database
Source: Sci Rep. 2024 Sep 12;14:21356. doi: 10.1038/s41598-024-72505-w (PMC11393075; doi:10.1038/s41598-024-72505-w)
Supplement: Supplementary file 1 — Supplementary Tables. [file 41598_2024_72505_MOESM1_ESM.docx]

**Table S1.** Generic names of all drugs used in the analysis.

| Category | Generic name |
| --- | --- |
| Active vitamin D_3_ analogs | Eldecalcitol, Alfacalcidol, Calcitriol |
| NSAIDs | Alminoprofen, Amfenac Sodium Hydrate, Aminopyrine, Bendazac, Benzydamine Hydrochloride, Bromfenac Sodium Hydrate, Bufexamac, Choline salicylate, Felbinac, Felbinac ethyl, Fenbufen, Fenoprofen calcium, Floctafenine, Flufenamic acid, Flurbiprofen axetil, Ibuprofen L-lysine, Ibuprofen Piconol, Diclofenac Sodium, Indometacin, Indometacin Farnesil, Indometacin Sodium Hydrate, Isopropylantipyrine, Ketophenylbutazone, Acemetacin, Proglumetacin Maleate, Sulindac, Etodolac, Piroxicam, lornoxicam, Meloxicam, Mesalazine, Migrenin, Mofezolac, Nepafenac, Nifenazone, Methyl Salicylate, Protizinic Acid, Salazosulfapyridine, Salicylamide, Sodium Salicylate, Sulpyrine Hydrate, Suprofen, Tenoxicam, Tiaprofenic Acid, Tolfenamic Acid, Ufenamate, Zaltoprofen, Ampiroxicam, Ibuprofen, Naproxen, Ketoprofen, Flurbiprofen, Oxaprozin, Loxoprofen Sodium Hydrate, Pranoprofen, Mefenamic Acid, Flufenamate aluminum, Celecoxib, Nabumetone, Ethenzamide, Tiaramide Hydrochloride, Metamizole sodium, Alclofenac, Fenbufen, Bromfenac, Amfenac, Tolmetin sodium, Esflurbiprofen, Diflunisal, Bucolome, Emorfazone, Epirizole |
| RASIs | Captopril, Enalapril Maleate, Lisinopril Hydrate, Perindopril Erbumine, Quinapril hydrochloride, Benazepril Hydrochloride, Aliskiren Fumarate, Cilazapril hydrate, Trandolapril, Delapril Hydrochloride, Temocapril Hydrochloride, Imidapril Hydrochloride, Alacepril, Azilsartan, Losartan Potassium, Valsartan, Irbesartan, Candesartan Cilexetil, Telmisartan, Olmesartan Medoxomil, Sacubitril Valsartan Sodium Hydrate |
| Loop diuretics | Furosemide, Torasemide, Azosemide, Bumetanide, Etacrynic Acid, Piretanide |
| Thiazide and thiazide-related diuretics | Hydrochlorothiazide, Trichlormethiazide, Benzylhydrochlorothiazide, Polythiazide, Cyclopenthiazide, Methyclothiazide, Penflutizide, Chlorthalidone, Mefruside, Clofenamide, Meticrane, Indapamide, Clorexolone, Tripamide |
| Magnesium oxide | Magnesium oxide |

NSAIDs, nonsteroidal anti-inflammatory drugs; RASIs, renin–angiotensin system inhibitors.

**Table S2.** Preferred terms list.

| Standardized MedDRA Query | PT code | PT |
| --- | --- | --- |
| Acute renal failure  Code: 20000003 | 10069339 | Acute kidney injury |
|  | 10069688 | Acute phosphate nephropathy |
|  | 10002847 | Anuria |
|  | 10003885 | Azotaemia |
|  | 10066338 | Continuous haemodiafiltration |
|  | 10061105 | Dialysis |
|  | 10078987 | Foetal renal impairment |
|  | 10018875 | Haemodialysis |
|  | 10053090 | Haemofiltration |
|  | 10049778 | Neonatal anuria |
|  | 10029155 | Nephropathy toxic |
|  | 10030302 | Oliguria |
|  | 10034660 | Peritoneal dialysis |
|  | 10072370 | Prerenal failure |
|  | 10038435 | Renal failure |
|  | 10038447 | Renal failure neonatal |
|  | 10062237 | Renal impairment |
|  | 10049776 | Renal impairment neonatal |
|  | 10081980 | Subacute kidney injury |
| Chronic kidney disease  Code: 20000213 | 10087686 | APOL1-mediated kidney disease |
|  | 10053699 | Artificial kidney device user |
|  | 10003885 | Azotaemia |
|  | 10064848 | Chronic kidney disease |
|  | 10078095 | Chronic kidney disease-mineral and bone disorder |
|  | 10010082 | Coma uraemic |
|  | 10012660 | Diabetic end stage renal disease |
|  | 10061105 | Dialysis |
|  | 10059015 | Dialysis device insertion |
|  | 10077512 | End stage renal disease |
|  | 10083258 | Erythropoietin deficiency anaemia |
|  | 10018367 | Glomerulonephritis chronic |
|  | 10018875 | Haemodialysis |
|  | 10053090 | Haemofiltration |
|  | 10019845 | Hepatorenal failure |
|  | 10062624 | High turnover osteopathy |
|  | 10020708 | Hyperparathyroidism secondary |
|  | 10023421 | Kidney fibrosis |
|  | 10063000 | Low turnover osteopathy |
|  | 10081588 | Metabolic nephropathy |
|  | 10058116 | Nephrogenic anaemia |
|  | 10067467 | Nephrogenic systemic fibrosis |
|  | 10029159 | Nephrosclerosis |
|  | 10049630 | Oedema due to renal disease |
|  | 10034498 | Pericarditis uraemic |
|  | 10034660 | Peritoneal dialysis |
|  | 10052279 | Renal and liver transplant |
|  | 10052278 | Renal and pancreas transplant |
|  | 10087816 | Renal artery revascularisation |
|  | 10038435 | Renal failure |
|  | 10074746 | Renal replacement therapy |
|  | 10038519 | Renal rickets |
|  | 10038533 | Renal transplant |
|  | 10056609 | Uraemia odour |
|  | 10046324 | Uraemic acidosis |
|  | 10087409 | Uraemic cardiomyopathy |
|  | 10046326 | Uraemic encephalopathy |
|  | 10063709 | Uraemic gastropathy |
|  | 10077910 | Uraemic myopathy |
|  | 10046328 | Uraemic neuropathy |
|  | 10060875 | Uraemic pruritus |
|  | 10067863 | Uridrosis |
| Hypertension  Code: 20000147 | 10000358 | Accelerated hypertension |
|  | 10005732 | Blood pressure ambulatory increased |
|  | 10005739 | Blood pressure diastolic increased |
|  | 10051128 | Blood pressure inadequately controlled |
|  | 10005750 | Blood pressure increased |
|  | 10063926 | Blood pressure management |
|  | 10053355 | Blood pressure orthostatic increased |
|  | 10005760 | Blood pressure systolic increased |
|  | 10081751 | Catecholamine crisis |
|  | 10063067 | Dialysis induced hypertension |
|  | 10012758 | Diastolic hypertension |
|  | 10014129 | Eclampsia |
|  | 10057615 | Endocrine hypertension |
|  | 10015488 | Essential hypertension |
|  | 10070538 | Gestational hypertension |
|  | 10049058 | HELLP syndrome |
|  | 10020571 | Hyperaldosteronism |
|  | 10020772 | Hypertension |
|  | 10049781 | Hypertension neonatal |
|  | 10059238 | Hypertensive angiopathy |
|  | 10020801 | Hypertensive cardiomegaly |
|  | 10058222 | Hypertensive cardiomyopathy |
|  | 10077000 | Hypertensive cerebrovascular disease |
|  | 10020802 | Hypertensive crisis |
|  | 10058179 | Hypertensive emergency |
|  | 10020803 | Hypertensive encephalopathy |
|  | 10079496 | Hypertensive end-organ damage |
|  | 10020823 | Hypertensive heart disease |
|  | 10055171 | Hypertensive nephropathy |
|  | 10058181 | Hypertensive urgency |
|  | 10049079 | Labile hypertension |
|  | 10025600 | Malignant hypertension |
|  | 10025603 | Malignant hypertensive heart disease |
|  | 10026674 | Malignant renal hypertension |
|  | 10026924 | Maternal hypertension affecting foetus |
|  | 10026985 | Mean arterial pressure increased |
|  | 10052066 | Metabolic syndrome |
|  | 10067598 | Neurogenic hypertension |
|  | 10089290 | Nocturnal hypertension |
|  | 10065508 | Orthostatic hypertension |
|  | 10076704 | Page kidney |
|  | 10050631 | Postoperative hypertension |
|  | 10036485 | Pre-eclampsia |
|  | 10065918 | Prehypertension |
|  | 10062886 | Procedural hypertension |
|  | 10087816 | Renal artery revascularisation |
|  | 10038464 | Renal hypertension |
|  | 10074864 | Renal sympathetic nerve ablation |
|  | 10038562 | Renovascular hypertension |
|  | 10038926 | Retinopathy hypertensive |
|  | 10039808 | Secondary aldosteronism |
|  | 10039834 | Secondary hypertension |
|  | 10084825 | Superimposed pre-eclampsia |
|  | 10078932 | Supine hypertension |
|  | 10088977 | Syndrome Z |
|  | 10042957 | Systolic hypertension |
|  | 10048007 | Withdrawal hypertension |
| Hyperglycaemia/new onset diabetes mellitus  Code: 20000041 | 10087376 | Acquired generalised lipodystrophy |
|  | 10089543 | Alpha hydroxybutyric acid increased |
|  | 10065367 | Blood 1,5-anhydroglucitol decreased |
|  | 10005557 | Blood glucose increased |
|  | 10012596 | Diabetes complicating pregnancy |
|  | 10012601 | Diabetes mellitus |
|  | 10012607 | Diabetes mellitus inadequate control |
|  | 10012631 | Diabetes with hyperosmolarity |
|  | 10077357 | Diabetic arteritis |
|  | 10012650 | Diabetic coma |
|  | 10080788 | Diabetic coronary microangiopathy |
|  | 10071265 | Diabetic hepatopathy |
|  | 10012668 | Diabetic hyperglycaemic coma |
|  | 10012669 | Diabetic hyperosmolar coma |
|  | 10012671 | Diabetic ketoacidosis |
|  | 10012672 | Diabetic ketoacidotic hyperglycaemic coma |
|  | 10012673 | Diabetic ketosis |
|  | 10074309 | Diabetic metabolic decompensation |
|  | 10081558 | Diabetic wound |
|  | 10080061 | Euglycaemic diabetic ketoacidosis |
|  | 10017395 | Fructosamine increased |
|  | 10072628 | Fulminant type 1 diabetes mellitus |
|  | 10018209 | Gestational diabetes |
|  | 10018429 | Glucose tolerance impaired |
|  | 10018430 | Glucose tolerance impaired in pregnancy |
|  | 10018478 | Glucose urine present |
|  | 10082836 | Glycated albumin increased |
|  | 10087214 | Glycated serum protein increased |
|  | 10018473 | Glycosuria |
|  | 10018475 | Glycosuria during pregnancy |
|  | 10018481 | Glycosylated haemoglobin abnormal |
|  | 10018484 | Glycosylated haemoglobin increased |
|  | 10085610 | Hepatogenous diabetes |
|  | 10020635 | Hyperglycaemia |
|  | 10087319 | Hyperglycaemic crisis |
|  | 10063554 | Hyperglycaemic hyperosmolar nonketotic syndrome |
|  | 10071394 | Hyperglycaemic seizure |
|  | 10071286 | Hyperglycaemic unconsciousness |
|  | 10056997 | Impaired fasting glucose |
|  | 10022489 | Insulin resistance |
|  | 10022491 | Insulin resistant diabetes |
|  | 10053247 | Insulin-requiring type 2 diabetes mellitus |
|  | 10023379 | Ketoacidosis |
|  | 10023388 | Ketonuria |
|  | 10023391 | Ketosis |
|  | 10023392 | Ketosis-prone diabetes mellitus |
|  | 10066389 | Latent autoimmune diabetes in adults |
|  | 10086189 | Maternally inherited diabetes and deafness |
|  | 10075980 | Monogenic diabetes |
|  | 10028933 | Neonatal diabetes mellitus |
|  | 10086425 | Neonatal hyperglycaemia |
|  | 10082630 | New onset diabetes after transplantation |
|  | 10033660 | Pancreatogenous diabetes |
|  | 10087435 | Pseudodiabetes |
|  | 10081755 | Steroid diabetes |
|  | 10067584 | Type 1 diabetes mellitus |
|  | 10067585 | Type 2 diabetes mellitus |
|  | 10072659 | Type 3 diabetes mellitus |
|  | 10057597 | Urine ketone body present |
| Cardiac failure  Code: 20000004 | 10063081 | Acute left ventricular failure |
|  | 10001029 | Acute pulmonary oedema |
|  | 10063082 | Acute right ventricular failure |
|  | 10007522 | Cardiac asthma |
|  | 10007554 | Cardiac failure |
|  | 10007556 | Cardiac failure acute |
|  | 10007558 | Cardiac failure chronic |
|  | 10007559 | Cardiac failure congestive |
|  | 10007560 | Cardiac failure high output |
|  | 10007625 | Cardiogenic shock |
|  | 10082480 | Cardiohepatic syndrome |
|  | 10051093 | Cardiopulmonary failure |
|  | 10068230 | Cardiorenal syndrome |
|  | 10063083 | Chronic left ventricular failure |
|  | 10063084 | Chronic right ventricular failure |
|  | 10084058 | Congestive hepatopathy |
|  | 10010968 | Cor pulmonale |
|  | 10010969 | Cor pulmonale acute |
|  | 10010970 | Cor pulmonale chronic |
|  | 10050528 | Ejection fraction decreased |
|  | 10051448 | Hepatojugular reflux |
|  | 10024119 | Left ventricular failure |
|  | 10024899 | Low cardiac output syndrome |
|  | 10049780 | Neonatal cardiac failure |
|  | 10073708 | Obstructive shock |
|  | 10037423 | Pulmonary oedema |
|  | 10050459 | Pulmonary oedema neonatal |
|  | 10076203 | Radiation associated cardiac failure |
|  | 10075337 | Right ventricular ejection fraction decreased |
|  | 10039163 | Right ventricular failure |
|  | 10060953 | Ventricular failure |

**Table S3.** Two-by-two contingency table for calculating the ROR.

|  | AKI (cases) | Adverse effects other than AKI (cases) |
| --- | --- | --- |
| Administered active vitamin D_3_ analogs | A | B |
| Administered other drugs | C | D |
| ROR =$\frac{\frac{A}{B}}{\frac{C}{D}}$ 95%CI=exp$\left[ \log\left( ROR \right)\pm1.96\sqrt{\frac{1}{A}+\frac{1}{B}+\frac{1}{C}+\frac{1}{D}} \right]$ | | |

AKI, acute kidney injury; ROR, reporting odds ratio.
